# Association between maternal folate status and gestational diabetes mellitus

**DOI:** 10.1002/fsn3.2173

**Published:** 2021-02-17

**Authors:** Yan Yang, Zixin Cai, Jingjing Zhang

**Affiliations:** ^1^ Department of Metabolism and Endocrinology Metabolic Syndrome Research Center Key Laboratory of Diabetes Immunology Ministry of Education National Clinical Research Center for Metabolic Diseases The Second Xiangya Hospital of Central South University Changsha China

**Keywords:** folate, GDM, gestational diabetes mellitus, meta‐analysis

## Abstract

Studies on the association between maternal folate status and gestational diabetes mellitus (GDM) have yielded inconsistent results. This meta‐analysis was performed to determine whether there may exist some association between maternal folate status and GDM. Unrestricted searches of PubMed, Web of Science, Cochrane, and Embase were conducted. All relevant studies on the association between maternal folat status and GDM risk were screened. The standardized mean difference (SMD) with 95% CIs was used to determine the association between maternal folate and GDM. Odds ratios (ORs) with 95% confidence intervals (CIs) were calculated using random‐effects models to assess the impact of maternal folate status on GDM risk. 12 studies were included. The overall data revealed that compared with the non‐GDM group, women with GDM had higher level of folate (SMD 0.41, 95% CI 0.07 to 0.21, *I^2^* = 17.2%) in second or third trimester. We also found that maternal high folate status may be associated with increased risk of GDM (OR 2.16, 95% CI 1.70 to 2.74, *I^2^* = 0.0%). Compared with non‐GDM group, women with GDM are prone to higher folate level. Moreover, high maternal folate status may predict a higher risk of GDM. As the number of included studies was limited, further large population studies are needed in the future.

## Graphical abstract

High folate status may predict a higher risk of GDM in pregnant women. However, more large studies are needed to confirm.

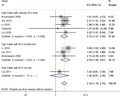

## 1 INTRODUCTION

Gestational diabetes mellitus (GDM), characterized by any degree of glucose intolerance that is first recognized during pregnancy, is a common complication of pregnancy, with an estimated incidence of approximately 17% globally (Amer, 2011; McIntyre et al., 2019). Accumulative evidence has shown that GDM plays a harmful role in the health of pregnant women and their offspring (Catalano et al., 2012; Damm et al., 2016). Regarding infants, maternal GDM increases the risk of adverse birth outcomes, including macrosomia, preterm birth, and large‐for‐gestational‐age (LGA) birth (Billionnet et al., 2017; Kong et al., 2019), as well as long‐term metabolic disorder (Tam et al., 2017). Regarding mothers, the risk of type 2 diabetes is 9.6 times greater for patients with GDM than for controls (Lee et al., 2007).

Folic acid (FA), a water‐soluble B vitamin, is now widely used to prevent neural tube defects (NTDs) in offspring (Bibbins‐Domingo et al., 2017), with a recommended daily dose of 400 micrograms (µg) from prepregnancy (4–12 weeks) until the end of the first trimester of pregnancy (8–12 weeks) in the majority (69.4%) of countries (Gomes et al., 2016). Surprisingly, many studies have found a link between maternal folate status and the development of GDM. However, studies have produced inconsistent conclusions regarding the association between maternal FA status and the risk of GDM (Cheng et al., 2019; Huang et al., 2019; Li, Zhang, et al., 2019; Zhu et al., 2016). A prospective cohort (*n* = 3,747) demonstrated a positive association between daily FA supplementation in the first trimester and the risk of GDM (Zhu et al., 2016). Consistent with this, high‐dose FA supplementation in early pregnancy was related to an increased risk of GDM in a Chinese cohort (*n* = 4,353) (Huang et al., 2019). However, a large prospective cohort (*n* = 20,199) indicated that prepregnancy habitual intake of FA supplementation was negatively associated with GDM risk in the USA (Li, Li, et al., 2019). Considering the inconsistent results mentioned above, determining the relationship between maternal FA status and GDM risk is urgent.

FA must be used during pregnancy to prevent NTDs in offspring, yet excessive use of FA may be associated with GDM risk (Cheng et al., 2019; Huang et al., 2019; Zhu et al., 2016). Together, these factors make it necessary to determine the role of maternal folate status in GDM development. This is the first meta‐analysis to explore the association between maternal folate level and GDM.

## 2 MATERIALS AND METHODS

### 2.1 Article search strategy

This meta‐analysis was rigorously projected according to the PRISMA (Preferred Reporting Items for Systematic Reviews and Meta‐Analyses) statement as described previously (Moher et al., 2009). The PROSPERO number is CRD42020191317. In this study, four electronic databases relevant to folate and GDM, including PubMed, Embase, Cochrane Library, and Web of Science, were searched. All published articles covering the relationship between folate and GDM were searched. The following search items were used: (“folic acid” OR “folate” OR “vitamin M” OR “vitamin B9” OR “B9, vitamin” OR “pteroylglutamic acid” OR “folvite” OR “folacin”) and (“GDM” OR “gestational diabetes” OR “diabetes, pregnancy‐induced” OR “diabetes, pregnancy induced” OR “pregnancy‐induced diabetes” OR “diabetes mellitus, gestational”). Additional papers were identified by manual searching and citation tracking.

### 2.2 Selection criteria

Two reviewers (YY and ZC) independently reviewed all the included studies, and the determination of eligible studies was finalized. Disagreements were settled through consensus or the help of a third reviewer (JZ). All the articles to be included in this meta‐analysis met the following criteria: (a) Studies with information on pregnancy folate status and GDM; (b) Studies with two groups, including a GDM and control group; and (c) Studies published in English. Articles were excluded if they met the following criteria: (a) Participants with multiple pregnancies or pre‐existing diabetes (type 1 diabetes or type 2 diabetes); (b) Meeting abstracts that contained insufficient information for assessment; or (c) Studies that did not have available information or usable data for the purpose of this meta‐analysis.

### 2.3 Data extraction

All relevant articles were guided in EndNote X9 software and reviewed independently by two authors (YY and ZC). Discrepancies between authors were settled with the help of a third reviewer (JZ). The following information was extracted by two independent investigators from the finalized study: author, year, country, study design, sample size, number of GDM, age (year), number of GDM cases, GDM criteria, and Newcastle–Ottawa Scale (NOS). All the extracted data were then imported into Excel software.

### 2.4 Quality assessment of studies

The quality of the included studies was assessed by the NOS (Stang, 2010). We assessed the quality of all relevant studies in accordance with the type of study, sample size, participant selection, representativeness of the sample, adequacy of follow‐up, comparability (exposed–unexposed or case–control), and method of ascertainment for cases and controls. A study with 6 or more scores was defined as high‐quality.

### 2.5 Definition of high folate status

So far, there is no accepted definition of high folate status. The World Health Organization (WHO) has recommended that RBC folate level greater than or equal to 906 nmol/L be the appropriate concentration for the prevention of neural tube defects (Cordero et al., 2015). Additionally, the first definition of serum high folate status was proposed by the WHO “based on the assay's upper limit capabilities without dilutions, and not on the biological implications for health” (WHO, 2015). Thus, there has not been a professional or biological cutoff for high folate status based on clinical outcomes or heath value, which necessitates determining the real definition of high folate status harmful to humans. In our included literature, the measured folate concentrations were divided into several categories according to various cutoff, and the incidence of GDM was then compared between the highest folate group and the lowest folate group. Considering that the criteria of high folate status are different in each study, we have added them to Table 3.

**TABLE 3:** Association between high folate status and GDM risk

ID	First author	Odds ratio and 95% CI	Folate status	Criteria of high folate status	Vitamin B12 status	Time for folate measurement	Time for GDM diagnose
1	Krishnaveni (Krishnaveni et al., 2009)	1.606 (0.609–4.234)	NA	NA	NA	third trimester	30 weeks
2	Krishnaveni (Krishnaveni et al., 2009)	2.125 (0.778–5.81)	NA	NA	B12‐insufficient	third trimester	30 weeks
3	Xie (Xie et al., 2019)	2.31 (1.51–3.52)	RBC folate, nmol/L (interquartile range, IQR)	(1,962.06–5,108.11)	NA	19–24 weeks (second trimester)	24–28 weeks
4	Lai (Lai et al., 2018)	1.97 (1.05–3.68)	plasma folate, nmol/L (Median, IQR)	(49.7, 44.5–58.5)	B12‐insufficient	26–28 weeks (third trimester)	26–28 weeks
5	Lai (Lai et al., 2018)	1.42 (0.61–3.3)	plasma folate, nmol/L (Median, IQR)	(49.7, 44.5–58.5)	B12‐normal	26–28 weeks (third trimester)	26–28 weeks
6	Li (Li, Hou, et al., 2019)	1.98 (1–3.9)	serum folate, ng/mL (Range of Values)	≥12.2	NA	24–28 weeks (third trimester)	24–28 weeks
7	Li (Li, Hou, et al., 2019)	3.08 (1.63–5.8)	serum folate, ng/mL (Range of Values)	≥12.2	B12‐insufficient	24–28 weeks (third trimester)	24–28 weeks
8	Sukumar (Sukumar et al., 2016)	1.12 (0.09–14.29)	serum folate, nmol/L (Range of Values)	(34.4–45.3)	NA	third trimester	26–28 weeks
9	Liu (Liu et al., 2020)	2.473 (1.013–6.037)	RBC folate (nmol/L)	RBC folate ≥ 862.5	NA	first trimester	24–28 weeks

### 2.6 Statistical analysis

All statistical analyses were performed using Stata (Version 13.0). The OR and 95% CI were used for discontinuous data. The heterogeneity among all studies was assessed by Q test and I2 statistics. All results were analyzed by a random‐effects model. Sensitivity analysis was performed by excluding each study one by one to evaluate the credibility of pooled results. Begg's and Egger's tests and funnel plots were used to detect potential publication bias, with a *p*‐value < .05 suggesting the presence of bias. The trim‐and‐fill method was further conducted to obtain an adjusted effect size when encountering publication bias.

## 3 RESULTS

### 3.1 Study characteristics

**FIGURE 1:**
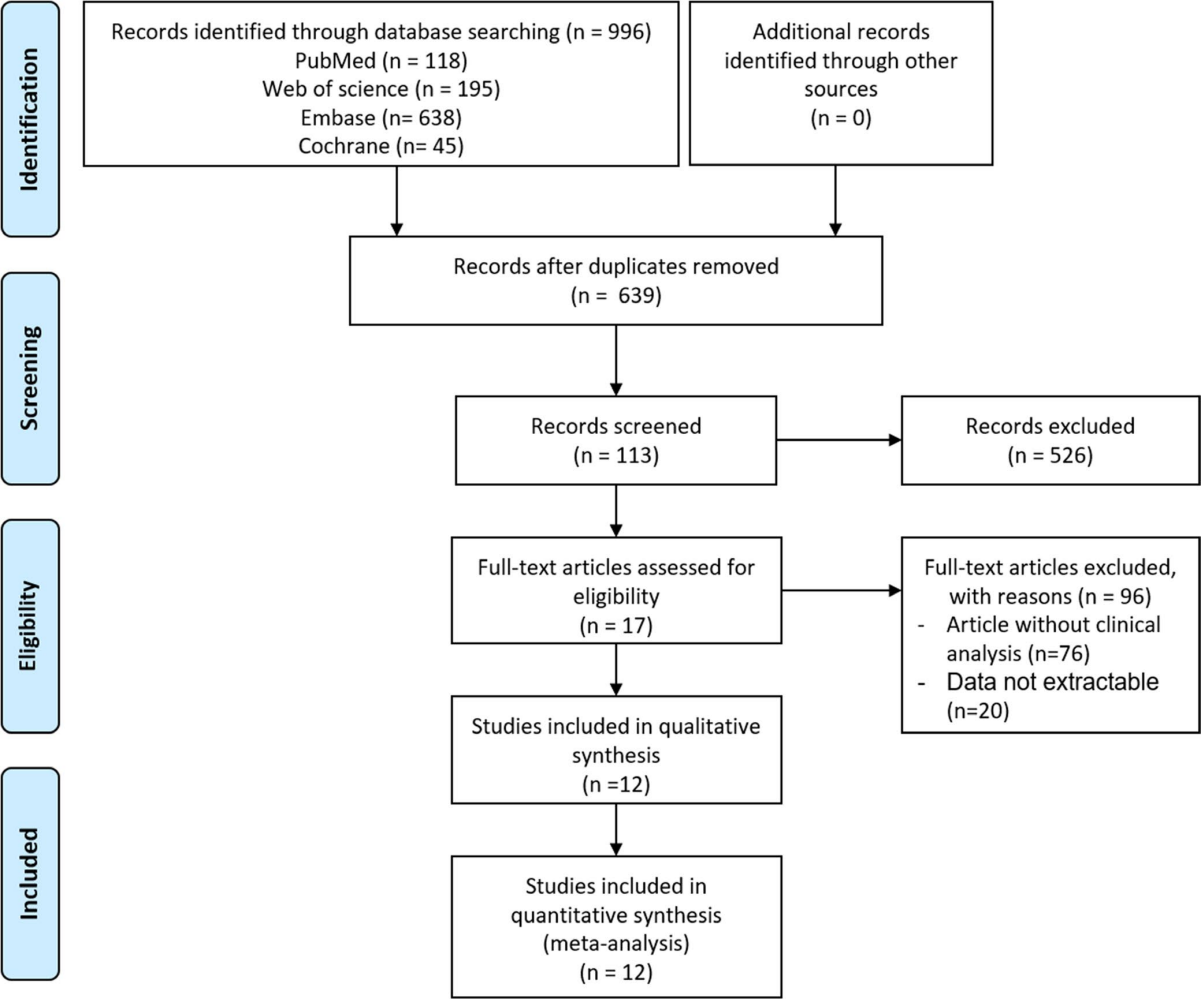
Flow diagram of the study selection process

The initial literature search (*n* = 996) on maternal folate status and GDM included searches in PubMed (*n* = 118), Web of Science (*n* = 195), Embase (*n* = 638), and Cochrane Library (*n* = 45) (Figure 1). No additional records were identified through other sources. In total, 357 duplications were excluded, and the titles and abstracts of the remaining 639 publications were screened. Overall, 526 publications were further eliminated, and 96 studies were subsequently excluded after full‐text screening. In the end, 17 studies met the selection criteria, 5 of which (Cheng et al., 2019; Huang et al., 2019; Li, Li, et al., 2019; Zhu et al., 2016) used FA supplement or intake instead of folate level as the standard of maternal folate status (Table S1). Therefore, we only included the remaining 12 studies (Barzilay et al., 2018; Berglund et al., 2016; Guven & Cetinkaya, 2005; Idzior‐Waluś et al., 2008; Krishnaveni et al., 2009; Lai et al., 2018; Li, Hou, et al., 2019; Liu et al., 2020; Seghieri et al., 2003; Sukumar et al., 2016; Tarim et al., 2004; Xie et al., 2019) for qualitative and quantitative (meta‐analysis) synthesis.

Among the 12 studies included in this analysis, there were 5,362 control subjects and 1,114 GDM patients. Three of the included studies were performed in China (Li, Hou, et al., 2019; Liu et al., 2020; Xie et al., 2019), 2 in Turkey (Guven & Cetinkaya, 2005; Tarim et al., 2004), 1 in Singapore (Lai et al., 2018), 1 in the UK (Sukumar et al., 2016), 1 in Spain (Berglund et al., 2016), 1 in Italy (Seghieri et al., 2003), 1 in India (Krishnaveni et al., 2009), 1 in Poland (Idzior‐Waluś et al., 2008), and 1 in the Canada (Barzilay et al., 2018) (Table 1). Regarding the criteria of GDM, four included studies used the Carpenter and Coustan or the American Diabetes Association (ADA) criteria, 2 studies used the WHO criteria, 1 used the International Association of Diabetes and Pregnancy Study Groups (IADPSG), 1 was based on criteria of National Diabetes Data Group (NDDG), and 2 used local criteria. The basic characteristics including results of quality assessment and the study design of the included studies are presented in Table 1.

**TABLE 1:** Description of eligible studies reporting the effect of maternal FA status on the risk of GDM

No.	Authors	Year	Country	Study design	Sample size	*N* GDM	Age,y	GDM criteria	NOS
1	Seghieri et al. (Seghieri et al., 2003)	2003	Italy	cross‐sectional	93	15	34.6 ± 3.1	the criteria suggested by the American Diabetes Association	6
2	Tarim et al. (Tarim et al., 2004)	2004	Turkey	Prospective cohort	304	28	32 ± 4.03	Carpenter and Coustan	6
3	Guven et al. (Guven et al., 2006)	2006	Turkey	cross‐sectional	223	30	30.0 ± 4.3	Carpenter and Coustan	7
4	Walus et al. (Idzior‐Waluś et al., 2008)	2008	Poland	cross‐sectional	61	44	30.5 ± 6.6	1999 World Health Organization criteria	8
5	Krishnaveni et al. (Krishnaveni et al., 2009)	2009	India	Prospective cohort	785	49	24	Carpenter and Coustan	6
6	Sukumar et al. (Sukumar et al., 2016)	2016	UK	Case–control	344	143	31.4 ± 5.8	The modified World Health Organization (WHO) 1999 criteria (fasting glucose ≥ 6.1 mmol/L or 2‐hr glucose ≥ 7.8 mmol/L) during the study period	7
7	Berglund et al. (Berglund et al., 2016)	2016	Spain	Prospective cohort	331	76	33.7 ± 4.6	the National Diabetes Data Group criteria and the Third International Workshop‐Conference on Gestational Diabetes Mellitus	7
8	Lai J et al. (Lai et al., 2018)	2018	Singapore	Prospective cohort	913	164	30.6 ± 5.2	1999 World Health Organization criteria	8
9	Barzilay et al. (Barzilay et al., 2018)	2018	Canada	Prospective cohort	368	16	34.4 ± 5.3	Canadian Diabetes Association 2008 practice guidelines	6
10	Li S et al. (Li, Hou, et al., 2019)	2019	China	cross‐sectional	406	90	29.4 ± 4.5	the International Association of Diabetes and Pregnancy Study Groups	9
11	Xie K et al. (Xie et al., 2019)	2019	China	Prospective cohort	2,282	392	27.89 ± 3.08	fasting glucose ≥ 5.5 mmol/L or a 2‐hr plasma glucose ≥ 8.0 mmol/L following a 75‐g oral glucose tolerance test at 24–28 weeks of gestation	9
12	Liu P et al (Liu et al., 2020)	2020	China	Prospective cohort	366	67	30.5 ± 4.0	the International Association of Diabetes and Pregnancy Study Groups	8

### 3.2 Association between maternal folate status and GDM

**FIGURE 2:**
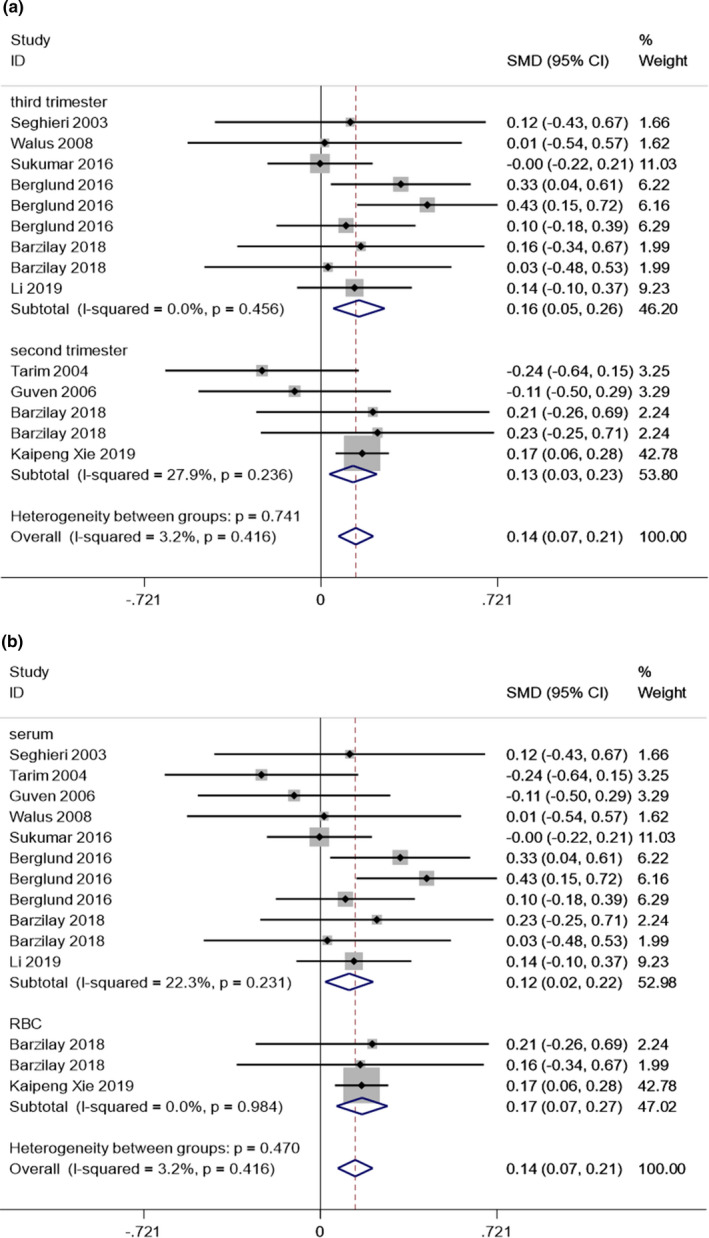
Forest plot for overall and subgroup analysis of standardized mean difference in folate levels between GDM cases and controls. (A) Subgroup analysis based on time for folate measurement (second or third trimester). (B) Subgroup analysis based on measurement folate status (serum or RBC folate)

As an indicator of maternal FA status, studies focusing on the concentration of maternal folate in GDM were analyzed. Nine studies were eligible for this part; among them, 14 results could be extracted to explore whether there was an association between maternal folate status and GDM (Table 2). Among the included nine studies, most of them performed GDM diagnosis during 24–28 weeks, with 3 exceptions. For comparison, all units of folate concentration were converted to nmol/L and all data presented as the medians and interquartile range were converted to the means and standard deviation according to the scenario 3 provided by Wan (Wan et al., 2014). The overall data showed that compared with the normal glucose tolerance, the folate concentration in second or third trimester was slightly higher in women who developed GDM (random‐effects pooled SMD [95% CI] 0.14 [0.07 to 0.21]; *I* ^2^ = 17.2%; Figure 2). Besides, two subgroup analyses were further conducted on the basis of measurement folate status (serum or RBC) and time for folate measurement (second or third trimester), while all the subgroup did not change the inverse association between folate status and GDM (Figure 2a‐b). The results for Egger's test (*p* = .512), Begg's test (*p* = .511), and funnel plot showed no publication bias (Figure S1a). Moreover, sensitivity analyses also demonstrated that our results were stable (Figure S1b).

**TABLE 2:** Comparison between GDM and non‐GDM women according to folate level

ID	First author	Folate level (nmol/L)		*p* value	Folate status	Time for folate measurement	Time for GDM diagnose
		Normal glucose tolerance	GDM				
1	Seghieri (Seghieri et al., 2003)	31.3 ± 16.5	33.3 ± 17.9	>.05	serum folate	24–28 weeks (third trimester)	24–28 weeks
2	Tarim (Tarim et al., 2004)	25.1 ± 11	22.5 ± 9.1	>.05	serum folate	24–28 weeks (third trimester)	24–28 weeks
3	Guven (Guven et al., 2006)	15.1 ± 7.2	14.36 ± 5	>.05	serum folate	24–28 weeks (third trimester)	24–28 weeks
4	Walus (Idzior‐Waluś et al., 2008)	25.1 ± 13.3	25.3 ± 13.5	>.05	serum folate	26–32 weeks (third trimester)	26–32 weeks
5	Sukumar (Sukumar et al., 2016)	23.17 ± 15.73	23.23 ± 14.86	>.05	serum folate	24–36 weeks (third trimester)	24–36 weeks
6	Berglund (Berglund et al., 2016)	34.77 ± 13.27	38.87 ± 11.33	.212	serum folate	24 weeks (third trimester)	24–34 weeks
7	Berglund (Berglund et al., 2016)	32.4 ± 13.1	38.1 ± 13.2	<.001	serum folate	34 weeks (third trimester)	24–34 weeks
8	Berglund (Berglund et al., 2016)	31.3 ± 15.5	32.9 ± 16.0	.001	serum folate	Delivery (third trimester)	24–34 weeks
9	Barzilay (Barzilay et al., 2018)	2,474 ± 526	2,586 ± 586	.43	RBC folate	12–16 weeks (second trimester)	24–28 weeks
10	Barzilay (Barzilay et al., 2018)	45.3 ± 39.5	46.4 ± 28.5	.66	serum folate	37–42 weeks (third trimester)	24–28 weeks
11	Barzilay (Barzilay et al., 2018)	59.8 ± 44.2	70.1 ± 52.6	.4	serum folate	12–16 weeks (second trimester)	24–28 weeks
12	Barzilay (Barzilay et al., 2018)	2,860 ± 610	2,962 ± 886	.69	RBC folate	37–42 weeks (third trimester)	24–28 weeks
13	Li (Li, Hou, et al., 2019)	21.7 ± 13.55	23.6 ± 15.07	.337	serum folate	24–28 weeks (third trimester)	24–28 weeks
14	Xie (Xie et al., 2019)	1,489.713 ± 549.81	1581.52 ± 527.88	.001212	RBC folate	19–24 weeks (second trimester)	24–28 weeks

### 3.3 Association between maternal high folate status and GDM risk

**FIGURE 3:**
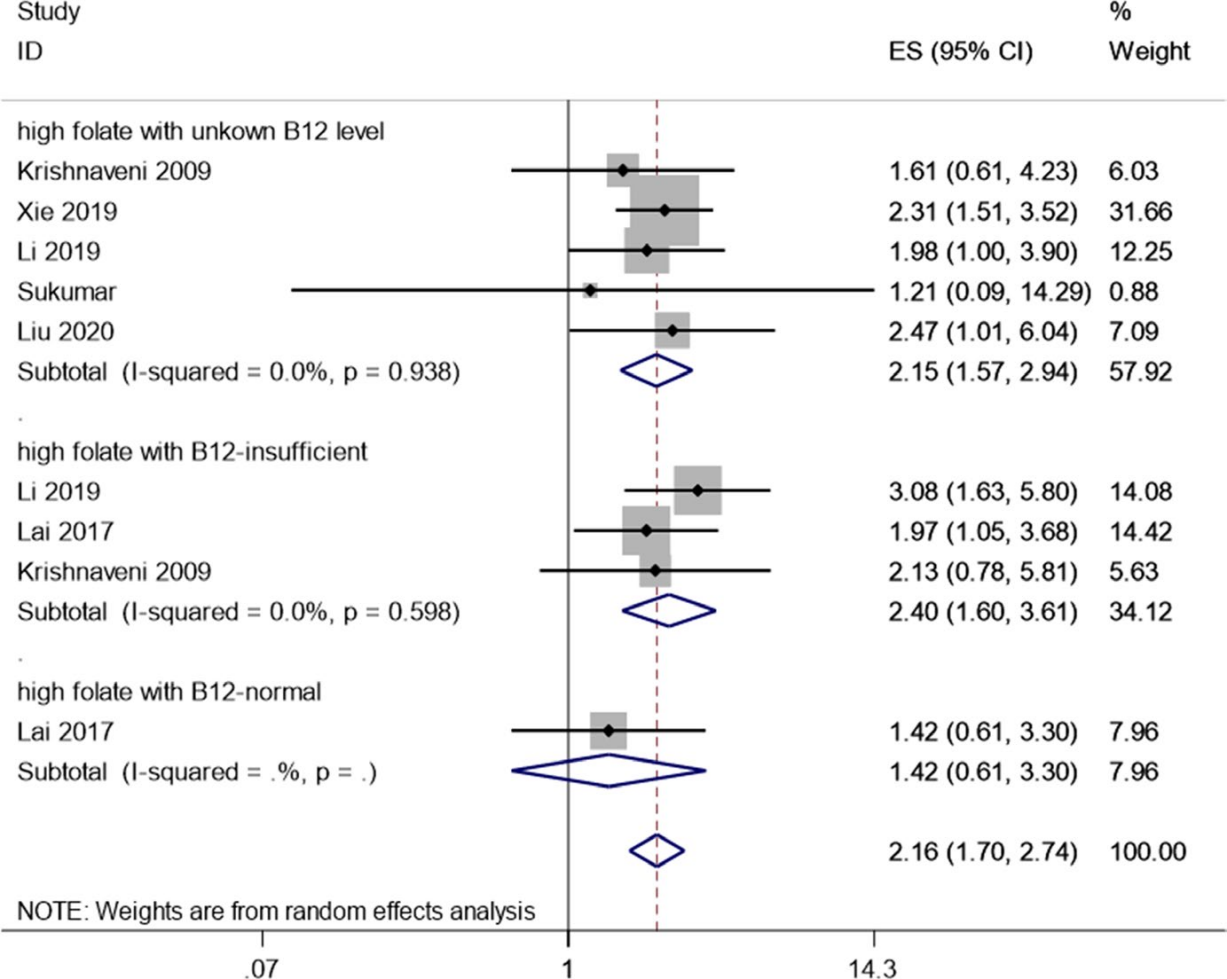
Forest plot for subgroup analysis (based on vitamin B12 status) of association between high folate status and GDM risk

Six studies were included for this part; among them, 9 results could be extracted to explore the effects of high maternal folate status on the risk of GDM (Table 3). Since most of the studies in this section refer to vitamin B12, the subgroup based on vitamin B12 was analyzed in this part. The overall results showed that high maternal folate was associated with higher GDM risk (random‐effects pooled OR [95% CI] 2.16 [1.70 to 2.74]; Figure 3). The prespecified subgroup based on vitamin B12 (B12) level further lessened the heterogeneity with no observed heterogeneity in each group (Figure 3). The OR was higher in the group with high FA concentrations combined with insufficient vitamin B12 (OR 2.40, 95% CI 1.60 to 3.61) and lower (OR 2.15, 95% CI 1.57 to 2.94) in the group with high FA concentrations regardless of vitamin B12 level (Figure 3). In addition, high FA combined with normal vitamin B12 had no association with GDM risk (OR 1.42, 95% CI 0.61 to 3.30) (Figure 3). Both Begg's test (*p* = .348), Egger's test (*p* = .192) and a symmetric distribution funnel plots (Figure S2a) indicated no publication bias. Moreover, the results of sensitivity analysis demonstrated that our analysis was stable (Figure S2b).

## 4 DISCUSSION

Women with GDM have an increased risk for prenatal morbidity and a considerably elevated risk for T2DM after pregnancy (McIntyre et al., 2019). FA supplementation during prepregnancy and pregnancy is widely used to prevent NTDs worldwide (Atta et al., 2016), contributing to an increased maternal folate status in women (Patel & Sobczyńska‐Malefora, 2017). Recently, side effects caused by FA overuse or high folate status have raised concerns. To determine the association between high folate status and GDM, we first conducted this meta‐analysis that was pooled from 12 studies.

### 4.1 Association between maternal folate status and GDM

Compared with pregnant women with normal glucose tolerance, GDM women have higher levels of folate in the second or third trimester (Figure 2). Our findings also revealed a positive association between high maternal folate concentration and GDM risk (Figure 3). The results of our subgroup analysis of vitamin B12 levels demonstrated that the combination of a low vitamin B12 status and high folate concentration in pregnancy makes women prone to GDM, according to the results of our hierarchical comparison, and that the combination of high folate levels and a vitamin B12‐normal status may have no association with GDM risk (Figure 3). These results indicated that low vitamin B12 levels may be one of the primary mechanisms driving the effect of high folate status on GDM.

### 4.2 Underlying mechanisms of the effects of high folate status on GDM

Although the mechanism underlying the adverse effects of high folate status on the risk of GDM remains unclear, several factors have been proposed. First, unmetabolized folic acid (UMFA) in blood is associated with the dysfunction of NK cells (Bayer & Fraker, 2017; Troen et al., 2006), leading to the imbalance of immune function and participating in the process of diabetes (Bonamichi & Lee, 2017; Chiba et al., 2016).

Additionally, vitamin B12 along with folate is essential for one‐carbon metabolism, including the synthesis of lipids, protein, and DNA (Finer et al., 2014). Vitamin B12 deficiency prevents DNA synthesis by inhibiting the production of tetrahydrofolate, while impaired mitochondrial DNA synthesis is associated with insulin resistance in obese subjects (Zheng et al., 2015).

Another possible mechanism is that low vitamin B12 during pregnancy can contribute to the increased levels of adipose‐derived circulating microRNAs, which may participate in the development of insulin resistance (Adaikalakoteswari et al., 2017). Moreover, high folate and low vitamin B12 levels lead to elevated levels of homocysteine (Selhub et al., 2007), which have been observed among women with GDM compared with controls (Cho et al., 2005; Guven et al., 2006). Hyperhomocystinemia also plays a harmful role in pancreatic β‐cell metabolism and insulin secretion (Engel et al., 2014).

### 4.3 Implication for practice and research

This meta‐analysis may have both important clinical and research implications. First, we demonstrated that maternal folate status was higher in GDM pregnant women than normal, and high folate status was associated with increased GDM risk especially in B12‐insufficient conditions, indicating that understanding the role of abnormally high folate levels in pregnant women is necessary. On the research side, our meta‐analysis indicated that low B12 level may be the critical factor for the negative influence of high folate status. More research is needed to identify the synergistic effect of high folate and low B12 status on GDM. The optimal maternal folate levels should also be the focus of future research.

### 4.4 Limitations of our study

The limitations of our meta‐analysis are as follows. First, due to the limited number of included studies, the conclusion that high folate status may increase the risk of GDM should be interpreted with caution. Second, considering the crucial role of B12 in GDM development, the effect of high folate status on GDM risk may depend on low B12 level. More studies are needed to determine the effect of high folate and low B12 status in GDM. Third, there were variations in the measurement of folate concentration within the included studies. Fourth, most of the maternal folate levels were measured in the second or third trimester among all included studies, which cannot prove a causal relationship between high folate status and GDM. Finally, high‐quality studies are needed to determine whether high maternal folate status may be an independent risk factor of GDM.

## 5 CONCLUSIONS

In summary, our meta‐analysis shows that a high folate concentration may be related to an increased GDM risk, especially when combined with vitamin B12 deficiency, indicating that high FA concentrations combined with low vitamin B12 may be a novel marker for predicting GDM risk. However, more large and well‐designed studies are needed to prove this finding and explore the synergistic effect of high folate and low B12 status on GDM development.

## 6 STUDIES INVOLVING HUMAN SUBJECTS

Although the study involves human subjects, it is a meta‐analysis based on evaluating published research data. Therefore, no ethical issues are involved.

## CONFLICTS OF INTEREST

The authors declare no conflict of interest.

## **AUTHOR** **CONTRIBUTION**

Y. Y. and Z. C. involved in conceptualization and formal analysis. Z. C., J. Z., and Y. Y. involved in methodology and validation. Y. Y. involved in software, investigation, and writing—original draft preparation. J. Z. involved in resources, writing—review and editing, visualization, project administration, funding acquisition. Z. C. involved in data curation. Z. C. and J. Z. supervised the study. All authors have read and agreed to the published version of the manuscript.

## Supporting information

Fig S1a

Fig S1b

Fig S2a

Fig S2b

Table S1
