# Supplementary figures and images for: Association between maternal folate status and gestational diabetes mellitus
Source: Food Sci Nutr. 2021 Feb 17;9(4):2042–52. doi: 10.1002/fsn3.2173 (PMC8020922; doi:10.1002/fsn3.2173)

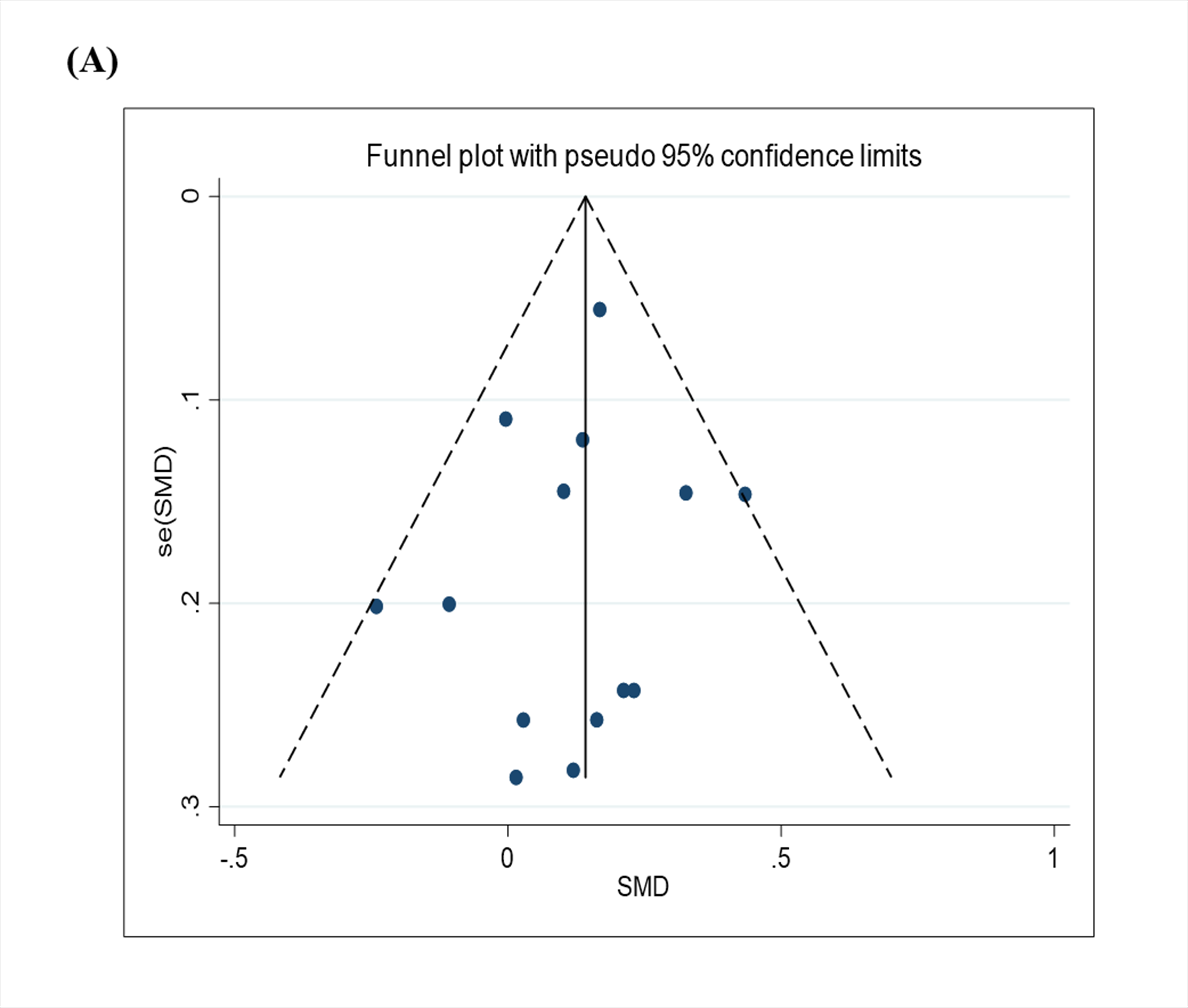

Supplement: Supplementary file 1 — Fig S1a [file FSN3-9-2042-s005.tif]

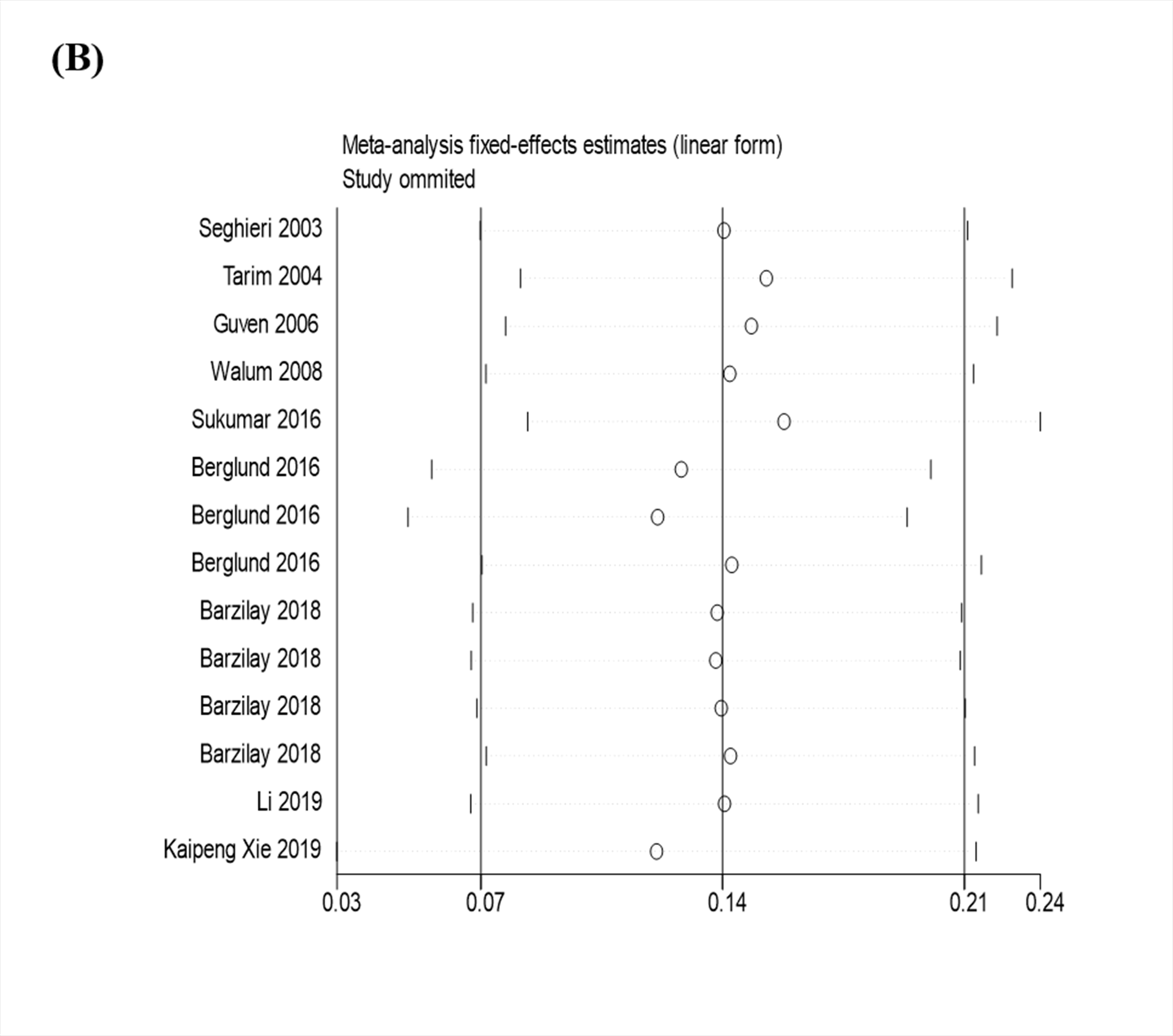

Supplement: Supplementary file 2 — Fig S1b [file FSN3-9-2042-s004.tif]

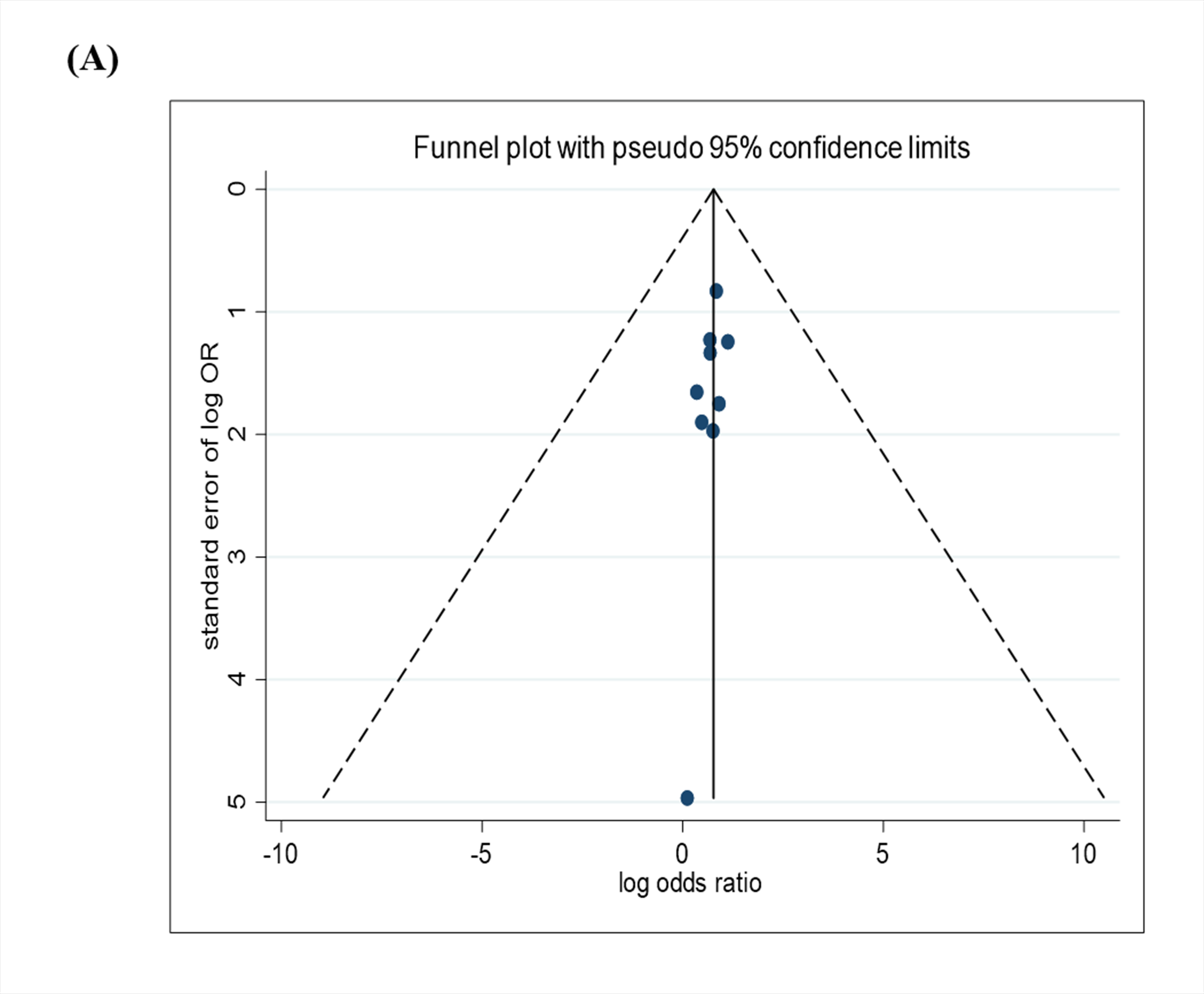

Supplement: Supplementary file 3 — Fig S2a [file FSN3-9-2042-s001.tif]

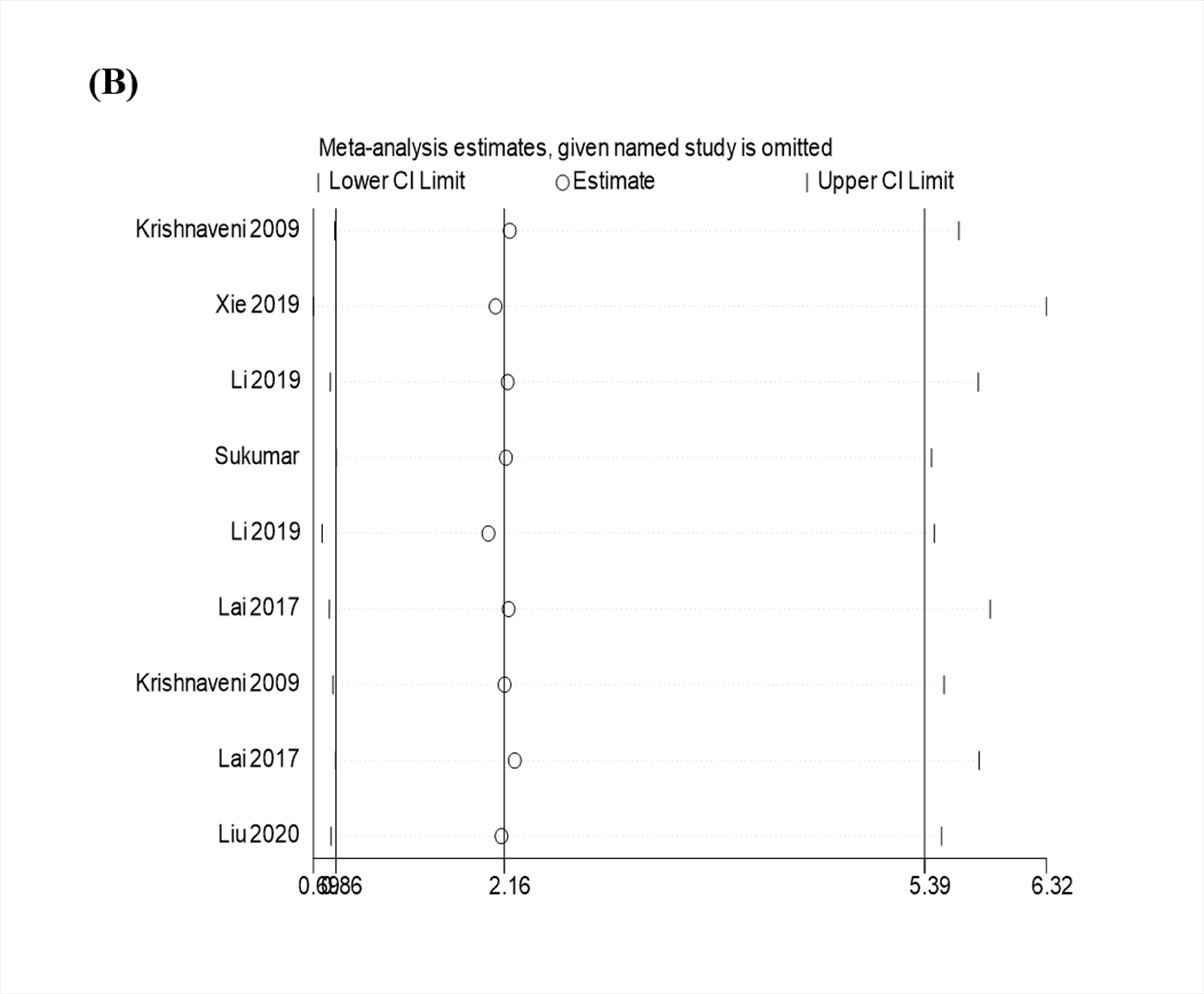

Supplement: Supplementary file 4 — Fig S2b [file FSN3-9-2042-s002.tif]
