# Supplementary material for: Association between maternal folate status and gestational diabetes mellitus
Source: Food Sci Nutr. 2021 Feb 17;9(4):2042–52. doi: 10.1002/fsn3.2173 (PMC8020922; doi:10.1002/fsn3.2173)
Supplement: Supplementary file 5 — Table S1 [file FSN3-9-2042-s003.docx]

**Supplementary Table 1:** Description of studies reporting the effect of FA supplementation on the risk of GDM.

| No. | Authors | Year | Country | Study design | Sample size | N GDM | Age,y | dose | FA–exposure  timing | Outcomes |
| --- | --- | --- | --- | --- | --- | --- | --- | --- | --- | --- |
| 1 | Zhu B et al. [13] | 2016 | China | cohort | 3474 | 249 | 26 | 400 ug/time | First trimester | daily FA supplement consumption in the first trimester was associated with an increased risk of GDM (adjusted odds ratio [OR] 2.25 [95% CI 1.35–3.76]). |
| 2 | Huang L et al. [12] | 2019 | China | cohort | 326 | 33 | 28.4±3.15 | In this study, the dose of FA supplementation ranged from 140 μg/d to 1200 μg/d. Approximately 93.5% of participants took FA at 400 μg/d. | 90-360d | There may be a positive association among prolonged folic acid supplementation, lipid profiles in the second trimester, and risk of GDM. |
| 3 | Li Q et al. [14] | 2019 | China | cohort | 4353 | 374 | NA | <400ug/d  400–800 ug/d  ≥800 ug/d | <150d, >150d | FA supplement use ≥800 mg/day from prepregnancy through midpregnancy was found to be associated with higher GDM risk. |
| 4 | Cheng G et al. [11] | 2019 | China | cohort | 950 | 97 | <25 25–29 30–34 35 | 400 ug/d | <90d, >90d | FA supplementation for 3 months before pregnancy was associated with an increased risk of GDM (adjusted relative risk (aRR): 1.72; 95% CI: 1.17–2.53). |
| 5 | Li M et al. [15] | 2019 | America | cohort | 14553 | 824 | 31.8 ±3.2 | 0ug/d 1‒399ug/d 400‒599ug/d ≥600ug/d | Prepregnancy | Higher habitual intakes of supplemental folate before pregnancy were significantly  associated with lower GDM risk. |
